# Oxytocin Modulates Microglial IL-17-Linked Inflammatory Pathways Through the IL-6/COX-2

**DOI:** 10.3390/life16010105

**Published:** 2026-01-12

**Authors:** Woochang Hwang, Yong Hun Jang, Juyoung Hong, Suyeon Kang, Junho K. Hur, Hyun Ju Lee

**Affiliations:** 1 Department of Pre-Medicine, College of Medicine, Hanyang University, Seoul 04763, Republic of Korea; whwang22@hanyang.ac.kr; 2 Hanyang Institute of Bioscience and Biotechnology, Hanyang University, Seoul 04763, Republic of Korea; 3 Department of Pediatrics, College of Medicine, Hanyang University, Seoul 04763, Republic of Korea; ryanjang93@hanyang.ac.kr; 4 Department of Biomedical Science, Graduate School of Biomedical Science and Engineering, Hanyang University, Seoul 04763, Republic of Korea; juyoung@hanyang.ac.kr (J.H.); oboe0912@gmail.com (S.K.); 5 Department of Genetics, College of Medicine, Hanyang University, Seoul 04763, Republic of Korea; 6 Department of Pediatrics, Hanyang University Hospital, Seoul 04763, Republic of Korea

**Keywords:** oxytocin, microglia, neuroinflammation, BV-2 microglial cells, IL-6, IL-17, COX-2

## Abstract

Neonatal neuroinflammation, driven by microglial activation and cytokine signaling, contributes to brain injury and adverse neurodevelopment outcomes. Perinatal inflammatory mediators, including interleukin-6, cyclooxygenase-2, and interleukin-17, prime microglia and influence circuit vulnerability. This study investigated whether oxytocin pretreatment attenuates lipopolysaccharide-induced inflammatory priming in BV-2 microglial cells. BV-2 microglia were preincubated with oxytocin (33 ng/mL) for 2 h, followed by lipopolysaccharide (0.5 µg/mL) for 2 h. Expression of ionized calcium-binding adapter molecule 1, a microglia marker, in BV-2 cells was assessed by immunofluorescence. After lipopolysaccharide treatment, the gene expression of BV-2 cells was assayed at 1, 2, and 6 h post stimulation by RT-qPCR and RNA-seq. Functional characterization of gene expression profile was performed. Analyses of gene expression profile of BV-2 cells by RT-qPCR and RNA-seq revealed that oxytocin pretreatment attenuated lipopolysaccharide-induced transcriptional activation, including interleukin-6 and cyclooxygenase-2 upregulation. Pathway enrichment analyses suggested that oxytocin-responsive genes were linked to the interleukin-17 signaling pathway. Gene Ontology enrichment analysis showed enrichment for genes related to cytokine production, membrane raft, and chemokine activity. Oxytocin pretreatment mitigates lipopolysaccharide-induced microglial activation by modulating the interleukin-17–interleukin-6/cyclooxygenase-2 axis, suggesting its potential role for oxytocin as an endogenous modulator of neuroinflammation during early brain development.

## 1. Introduction

Neonatal neuroinflammation is a central driver of acute brain injury and adverse neurodevelopmental trajectories in both preterm and term infants, arising from coordinated crosstalk among microglia, cytokine networks, and intracellular signaling pathways [1,2,3,4]. Inflammatory injury to the developing brain is a critical complication that can occur before, during, or after a brain injury, leading to direct cellular damage, disruption of neurodevelopmental processes, and long-term neurological sequelae such as cerebral palsy [5,6,7]. Perinatal infection, hypoxia–ischemia, or other stressors can trigger systemic upregulation of inflammatory cytokines and activation of resident immune cells known as microglia [5,8,9]. Microglia—the resident immune cells of the brain—initiate inflammatory programs that are adaptive when transient but become pathological when activation is prolonged or dysregulated, shifting toward an inflammatory primed state that exacerbates neuronal dysfunction through sustained release of pro-inflammatory cytokines, chemokines, and reactive oxygen species [2,10,11]. These inflammatory cascades extend beyond parturition, shaping neonatal organ injury—including that of the brain—and establishing trajectories of long-term neurodevelopmental risk [3,12,13,14].

In preterm infants, early inflammatory exposure can shift microglia toward a hyper-responsive inflammatory “primed” state that overreacts to subsequent stimuli and adopts a neurotoxic phenotype, resulting in persistent vulnerability to neurodevelopmental impairment [15,16,17]. Under normal conditions, microglia play essential roles in synaptic pruning, circuit refinement, and axonal guidance through complement-mediated synapse elimination; however, inflammation priming during these critical developmental windows can disrupt these processes and impair circuit maturation [18,19,20,21,22]. Consistent with this framework, neuroimaging studies in preterm cohorts have identified MRI biomarkers of microglia-linked neuroinflammatory injury, including reduced cortical gyrification, diminished cortical thickness, and delayed maturation of white-matter microstructure [23,24,25]. Neuroinflammatory processes associated with preterm birth are most strongly linked to white-matter injury and cerebral palsy, whereas perinatal conditions such as chorioamnionitis and systemic neonatal infection associated with increased risks of cognitive, motor, and language impairments [7,26,27,28].

Oxytocin (OT) is a hypothalamic nonapeptide produced in the paraventricular and supraoptic nuclei that signals through the widely distributed oxytocin receptor (OXTR) to regulate neuroendocrine function, autonomic tone, and socio-affective behavior [29,30]. In microglial inflammatory contexts, interleukin-17 (IL-17) receptor signaling can engage mitogen-activated protein kinase (MAPK) cascades, including extracellular signal-regulated kinase 1 and 2 (ERK1/2) and p38, and has been linked to inflammatory mediator induction [31], whereas emerging preclinical evidence indicate that OT exerts anti-inflammatory and antioxidant effects in microglia: OT pretreatment reduces lipopolysaccharide (LPS)-induced activation, suppresses pro-inflammatory mediator release, and inhibits ERK1/2 phosphorylation and p38 MAPK in an OXTR-dependent manner [30,32,33,34]. In perinatal inflammatory conditions, IL-17-associated inflammatory programs have been increasingly implicated in infection and intrauterine inflammation, and can converge on the induction of inflammatory mediators such as interleukin-6 (*IL-6*) and cyclooxygenase-2 (*COX-2*) [35]. Together, these findings suggest that OT may function as an endogenous regulator of microglial inflammatory priming in developmental contexts characterized by IL-17–linked inflammatory signaling. Microglia also express IL-17 receptors and can respond to IL-17–driven inflammatory cues, suggesting a potential inter-section between IL-17-linked outputs and microglial inflammatory priming during early neurodevelopment [36]. However, the mechanistic role of OT in preterm neuroinflammation—particularly regarding *IL-6*, *COX-2*, and IL-17-linked pathways—remains unclear. Although OT is not routinely administered to preterm infants, its potential neuroprotective effects under conditions of procedural pain and stress are being under active investigation [29,37]. In preterm infants, exposure to maternal voice and touch immediately before an inflammatory or painful trigger has been associated with activation of the infant OT system, as evidenced by lower pain scores and increased salivary OT concentration [38]. However, the clinical conditions under which OT-related interventions yield reproducible neuroprotective or anti-inflammatory benefits in preterm infants have not been clearly established. In practice, clinical findings remain inconsistent across studies [37], which likely reflects substantial heterogeneity in populations and study designs, along with variability in dosing regimens, formulation, route and duration of administration [37]. In addition, limited characterization of pharmacokinetics and pharmacodynamics in pediatric populations and the absence of validated downstream biomarkers to confirm delivery and target engagement continue to constrain cross-study comparability and mechanistic interpretation [39,40].

We therefore sought to examine how OT pretreatment modulates LPS-induced inflammatory responses in BV-2 microglia, integrating classical inflammatory readouts with transcriptomic profiling to evaluate OT as a physiological brake on microglial hypersensitivity. Based on previous studies, we anticipated that BV-2 cell line is an adequate in vitro model to investigate the effect of OT on the activation of primed microglia by LPS [41,42].

## 2. Materials and Methods

### 2.1. Cell Culture and Treatment 

We selected BV-2 cells as a model for primed or pre-activated microglia based on previous studies [41,42]. Based on the previous studies, we anticipated that the immortalized BV-2 cells may reflect the characteristics of resting microglia before treatment of LPS and change to activated state with LPS, although they are not perfect replicates of primary microglia. The BV-2 cells used in this study were kindly provided by Dr. Yun Kyung Kim’s laboratory at the Korea Institute of Science and Technology (KIST). BV-2 murine microglial cells were cultured in Dulbecco’s Modified Eagle Medium (DMEM) medium containing 10% Fetal bovine serum (FBS) and 1% penicillin–streptomycin, at 37 °C in a CO_2_ incubator. To check the BV-2 cells maintained the microglia characteristics, the expression of ionized calcium-binding adapter molecule 1 (*IBA1)*, a common microglia maker, was confirmed by RT-qPCR and immunofluorescence [43]. One hour before OT treatment, the culture medium was replaced with serum-free DMEM. Cells were preincubated with OT (33 ng/mL; 0.03 uM or 330 ng/mL; 0.3 uM) for 1, 2, or 6 h and subsequently stimulated with lipopolysaccharide (LPS; 0.5 µg/mL) for an additional 2 h under serum-free conditions. The oxytocin concentrations were selected based on a previous study, which demonstrated effective anti-inflammatory activity at similar doses [34]. For time-course experiments, cells were harvested 1, 2, and 6 h after LPS treatment to evaluate temporal changes in gene expression.

### 2.2. Immunofluorescence Staining

BV-2 cells were seeded on coverslips in 24-well plates and cultured for 24 h. The seeded cells were then washed with Dulbecco’s phosphate-buffered saline (DPBS) and fixed with 3.8% paraformaldehyde (pH 7.4) for 15 min. Next, the cells were permeabilized with 0.3% Triton X-100 and blocked with 2% normal goat serum [44] for 1 h at room temperature. Cells were then incubated overnight at 4 °C with rabbit anti-*IBA1* antibody (Abcam, Cambridge, UK, ab178846) diluted in 0.1% NGS, rinsed with DPBS, and incubated with Alexa Fluor™ 647–conjugated donkey anti-rabbit immunoglobulin G (IgG) secondary antibody (Invitrogen, Carlsbad, CA, USA, A-31573) for 1 h at room temperature. Cells were mounted with Antifade Mounting Medium containing 4′,6-diamidino-2-phenylindole (DAPI) (VECTASHIELD^®^, Newark, CA, USA, H-1200). Confocal images were acquired by a Zeiss LSM 900 microscope. Samples processed without primary antibody were used as negative controls to determine non-specific staining.

### 2.3. RNA Extraction and RT-qPCR

Total RNA was extracted using the Easy-Spin™ Total RNA Extraction Kit (iNtRON Biotechnology, Seongnam City, Korea) according to the manufacturer’s protocol. cDNA was prepared with the High-Capacity cDNA Reverse Transcription Kit (Applied Biosystems, Foster City, CA, USA). qPCR was performed with SYBR Green chemistries on a Bio-Rad CFX Connect Real-Time PCR Detection System (Optics Module) (Bio-Rad Laboratories, Inc., Hercules, CA, USA). The sequences of primers used for RT-qPCR are listed in Supplementary Table S1. *GAPDH* was used as the internal reference gene for quantifying the relative gene expression based on the observed Ct values.

### 2.4. Transcriptome Analysis by mRNA Sequencing

The quality of RNA extracted from BV-2 microglial cells were assessed using an Agilent TapeStation D1000 system (Waldbronn, Germany). RNAs that passed the quality score were utilized to prepare mRNA sequencing libraries using the NEBNext^®^ Ultra™ II RNA Library Prep Kit for Illumina^®^ (San Diego, CA, USA) according to the manufacturer’s protocol. Libraries were sequenced on an Illumina platform to generate 150 bp paired-end sequencing reads. The raw sequencing reads were quality-checked and trimmed prior to alignment. Trimmed sequence reads were aligned to Ensembl mouse genome version 38 (GRCm38) with Hierarchical Indexing for Spliced Alignment of Transcripts 2 (HISAT2). RNA-seq experiments were performed in biological triplicates (n = 3 per group). Gene expression levels were quantified using htseq-count, and genes with a total read count of <60 across the two comparison groups (three replicates per group) were excluded from differentially expressed genes (DEGs) analysis. DEGs were determined using DESeq2 R package (version 1.48.2) with Benjamini–Hochberg adjusted *p* values and log2 fold changes. We defined DEGs as those with an adjusted *p*-value < 0.05 and an absolute log2 fold change > 1 (i.e., log2 fold change < −1 or >1).

### 2.5. KEGG Pathway and Gene Ontology Enrichment Analysis

Mouse gene annotation was performed using org.Mm.eg.db v3.21.0. DEGs with adjusted *p*-value below 0.05 were subjected for enrichment analysis using clusterProfiler R package (version 4.14.6). Kyoto Encyclopedia of Genes and Genomes (KEGG) pathways were retrieved from KEGG release 116.0 (1 October 2025). Gene Ontology (GO) enrichment was conducted for the Biological Process (BP), Molecular Function (MF), and Cellular Component (CC) ontologies. The *p* values were adjusted using the Benjamini–Hochberg method. Dot plots summarize the GeneRatio, adjusted *p* values, and term gene counts.

### 2.6. Statistical Analyses

All experiments were conducted with two or more independent biological replicates. Differences in the gene expression levels were analyzed using Student’s *t*-test. Error bars in the box plots indicate standard error of the mean (SEM).

## 3. Results

### 3.1. Effect of Oxytocin Pretreatment in Immune Response of BV-2 Cells to Lipopolysaccharide

**Figure 1:**
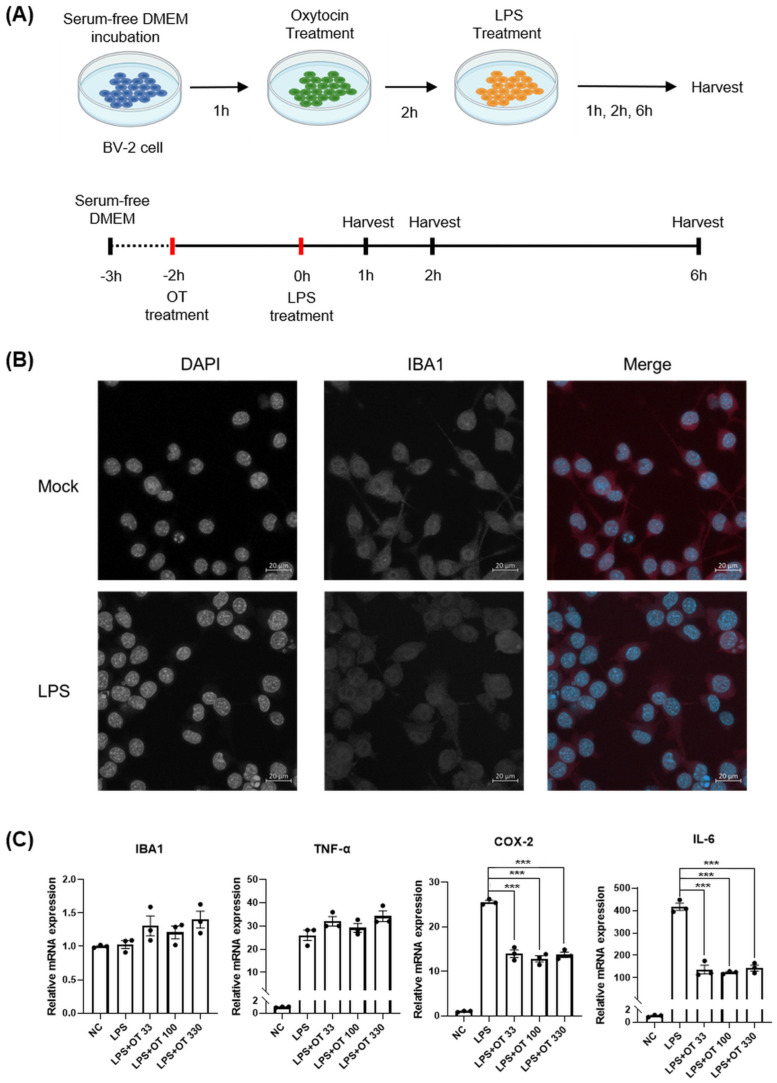
Experimental design and microglial activation following LPS stimulation. (**A**) Schematic illustration of the experimental workflow. BV-2 cells were pretreated with oxytocin, and subsequently stimulated with LPS. Cells were harvested at 1, 2, or 6 h for gene expressions analysis. (**B**) *IBA1* immunofluorescence in mock and LPS-treated BV-2 cells. Cells were stimulated with LPS (0.5 µg/mL). Scale bar = 20 µm. (**C**) Validation of oxytocin effects on LPS-induced inflammatory responses in BV-2 cells. RT-qPCR analysis of *IBA1*, *TNF-α*, *COX-2*, and *IL-6* expression under NC, LPS alone, and LPS with OT at different concentrations (33, 100, and 330 ng/mL). Data are presented as mean ± SEM (n = 3), internally normalized to GAPDH renormalized to NC. ***, *p* < 0.0001. Abbreviations: BV-2, BV-2 microglial cell line; *COX-2*, cyclooxygenase-2; DAPI, 4′,6-diamidino-2-phenylindole; DMEM, Dulbecco’s Modified Eagle Medium; DPBS, Dulbecco’s phosphate-buffered saline; GAPDH, glyceraldehyde 3-phosphate dehydrogenase; *IBA1*, ionized calcium-binding adapter molecule 1; *IL-6*, interleukin-6; LPS, lipopolysaccharide; NC, negative control; OT, oxytocin; SEM, standard error of the mean; *TNF-α*, tumor necrosis factor alpha.

To assess the modulatory effect of OT on microglial inflammatory responses to LPS, we sought to analyze the changes in gene expression patterns in BV-2 microglia cells in response to OT and LPS treatment (Figure 1A).

To this end, we first established a primed microglial state without provoking excessive inflammatory activation. We conducted immunofluorescence to observed that BV-2 cells showed robust expression levels of *IBA1*, a microglia marker, consistent with the glial characteristics (Figure 1B; Supplementary Figure S1). Immunofluorescence analyses showed that *IBA1* expression remained consistent, suggesting that the BV-2 cells maintained the glial cell characteristics after treatment of a range of LPS doses (0.1–100 μg/mL). We observed that the *IBA1* expression levels in BV-2 cell remained stable within the concentrations of LPS (Supplementary Figure S1). Based on the results, we selected 0.5 μg/mL of LPS concentration for inducing LPS-dependent transcriptional priming.

To assess how OT may affect the response of BV-2 cells to LPS, we analyzed the gene expression levels of *IBA1*, Tumor necrosis factor alpha (*TNF-α*), *COX-2*, and *IL-6* in BV-2 cells that were pretreated with OT (33, 100, and 330 ng/mL) for 2 h prior to LPS (0.5 µg/mL) stimulation (Figure 1C). After LPS stimulation, RNA from the cells were harvested at 1, 2, and 6 h for downstream analyses (Figure 1C; Supplemental Figure S2A,B). We conducted RT-qPCR to quantify the expression levels of *IBA1*, *TNF-α*, *COX-2*, and *IL-6* under the five experimental conditions: negative control (NC), LPS alone, and LPS with OT pretreatment at 33, 100, or 330 ng/mL (Figure 1C). Compared with NC, LPS treatment increased three markers: *TNF-α*, *COX-2*, and *IL-6* showed marked elevations. On the other hand, *IBA1* expression levels remained relatively stable across all groups, with only minor variation around baseline levels and no statistically significant differences among conditions. At 1 h, comparison between LPS and LPS + OT at all OT concentrations tested showed a significant reduction in *COX-2* and *IL-6* expression following OT pretreatment (*p* < 0.0001). *IBA1* and *TNF-α* expression levels did not differ significantly between these two groups. The results suggested that OT pretreatment partly blunted the LPS response: *COX-2* and *IL-6* were significantly reduced, while *IBA1* and *TNF-α* expression levels were essentially unchanged.

We next sought to ask whether these transcriptional effects persisted over time, the identical experimental conditions were applied at 2 and 6 h post-stimulation time points (Supplementary Figure S2A,B). We found that, within the 2 and 6 h time frame, the OT-associated effect of reducing *COX-2* and *IL-6* expressions seemed to be sustained. Consistent with the 1 h results, *IBA1* displayed a similar expression level irrespective of OT pretreatment.

### 3.2. Transcriptome Analyses Under OT Pretreatment

**Figure 2:**
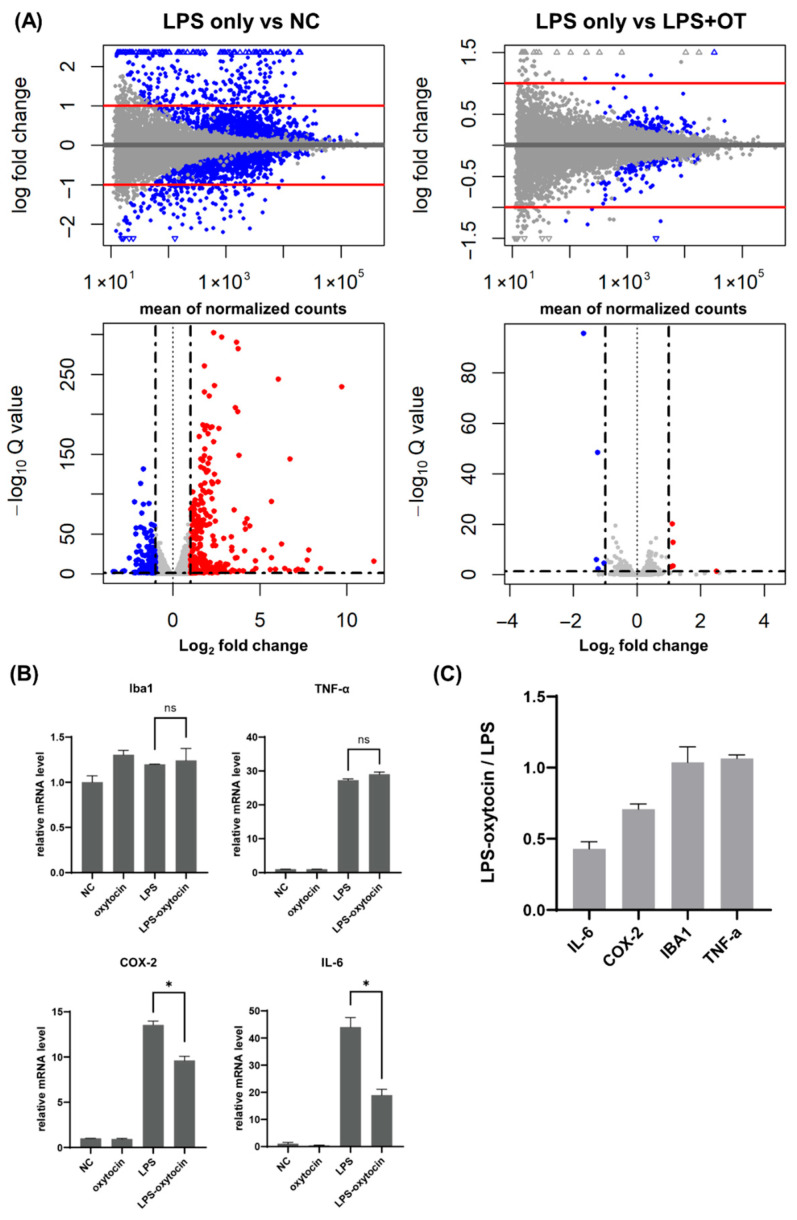
RNA-seq analysis demonstrates that oxytocin attenuates LPS-induced inflammatory responses in BV-2 microglia. (**A**) RNA-seq of BV-2 cells treated with NC, OT (33 ng/mL), LPS (0.5 µg/mL, 2 h), and LPS + OT. MA and volcano plots are shown for two contrasts: LPS vs. NC and LPS vs. LPS + OT. Differentially expressed genes were defined as adjusted *p* < 0.05 and |log2FC| ≥ 1 (red, upregulated; blue, downregulated; gray, not significant). (**B**) Normalized RNA-seq expression of *IBA1*, *IL-6*, *TNF-α* and *COX-2* under NC, OT, LPS, and LPS + OT conditions. Oxytocin reduced IL-6 and *COX-2*, whereas *IBA1* and *TNF-α* showed no significant change. OT denotes oxytocin (33 ng/mL). (**C**) Relative mRNA expression changes in *IBA1*, *IL-6*, *TNF-α*, and *COX-2* in BV-2 cells. *, *p* < 0.05. Abbreviations: BV-2, BV-2 microglial cell line; *COX-2*, cyclooxygenase-2; FC, fold change; *IBA1*, ionized calcium-binding adapter molecule 1; *IL-6*, interleukin-6; LPS, lipopolysaccharide; NC, negative control; OT, oxytocin; *TNF-α*, tumor necrosis factor alpha.

Next, we examined how OT pre-treatment modulates the RNA expression profiles following LPS treatment. To this end, we conducted RNA sequencing of BV-2 cells after treatment of LPS, with or without OT pre-incubation (Figure 2A). RNA integrity and library quality were first confirmed using the Agilent TapeStation D1000 system, which showed clear peaks between 200 and 400 bp across all samples, consistent with high-quality library preparation (Supplementary Figure S3).

The transcriptome analyses showed that LPS treatment resulted in overall changes in gene expression profile of BV-2 cells compared to non-treated condition. In the LPS vs. NC contrast, a broad set of genes was significantly up- or downregulated, producing a wide vertical spread on MA/volcano plots with many transcripts surpassing log_2_ fold-change and adjusted Q-value thresholds.

As the RT-qPCR analyses showed that the upregulation of *COX-2* and *IL-6* could be suppressed by OT, we sought to ask what genes were regulated by OT treatment after LPS stimulation. To this end, we compared the RNA-seq data between LPS-only and OT-LPS conditions and found a group of genes that were decreased by pretreatment of OT before LPS stimulation (Figure 2A). In contrast to the LPS vs. NC analyses, the LPS vs. LPS + OT comparison revealed only a small subset of DEGs, with a visible narrowed log_2_ fold-change range and reduced dispersion along the Q-value axis. Most transcripts remained close to baseline, showing that OT pretreatment contracted both the amplitude and the statistical strength of the LPS-driven proinflammatory transcriptome. In summary, LPS inherently elicits a broad, high-intensity proinflammatory transcriptome, whereas OT pretreatment constrains this program by reducing both its breadth and its amplitude, consistent with attenuation of a subset of LPS-responsive transcripts.

Expression levels of the representative inflammatory genes (*IL-6*, *COX-2*, *IBA1*, and *TNF-α*) were extracted from the RNA-seq data and visualized in Figure 2B. *IL-6* and *COX-2* were robustly induced by LPS, with mRNA levels markedly higher than in control or OT-only cultures, but both were clearly reduced in the LPS + OT condition. This directionality was consistent with the RT-qPCR results for *IL-6* and *COX-2*. By contrast, *IBA1* and *TNF-α* showed no significant differences between LPS only and LPS + OT and remained comparable across these two groups, indicating lower sensitivity to OT pretreatment under the same conditions. Consistently, normalized expression relative to LPS only group demonstrated that *IL-6* and *COX-2* were more strongly downregulated than *TNF-α* and *IBA1* in the LPS + OT group, indicating a selective dampening of LPS-induced inflammatory transcripts (Figure 2C).

### 3.3. Pathway Enrichment and Orthogonal Validation

**Figure 3:**
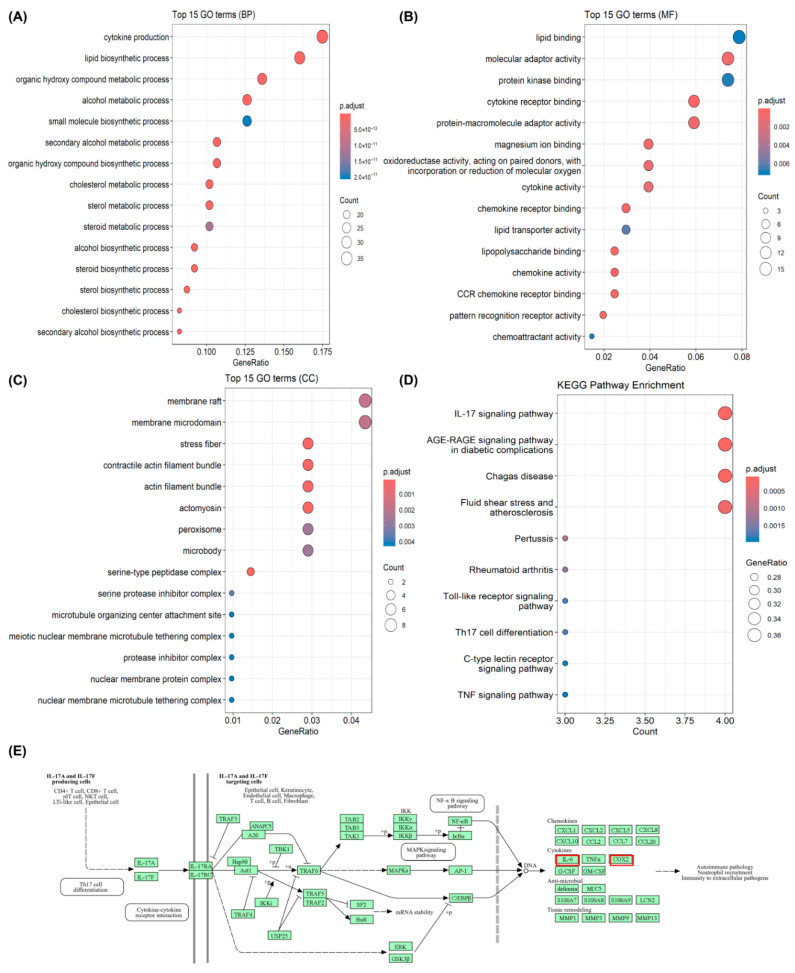
RNA-seq analysis reveals oxytocin-mediated transcriptional changes in BV-2 microglia. (**A**) GO enrichment analysis (Biological Process, BP) of DEGs from LPS + OT versus LPS. Top enriched biological processes are displayed (dot size, gene counts; color, adjusted *p* value; x-axis, GeneRatio). (**B**) GO enrichment analysis (Molecular Function, MF) of DEGs from the same comparison. Top enriched molecular functions are displayed (dot size, gene counts; color, adjusted *p* value; x-axis, GeneRatio). (**C**) GO enrichment analysis (Cellular Component, CC) of DEGs from the same comparison. Top enriched cellular components are displayed (dot size, gene counts; color, adjusted *p* value; x-axis, GeneRatio). (**D**) KEGG pathway enrichment analysis of downregulated genes in LPS + OT compared with LPS (log2FC < −0.7, *p*-adj < 0.05) identifying the IL-17 signaling pathway as the most significantly affected. (**E**) Schematic representation of the IL-17 signaling pathway adapted from KEGG. Key downstream targets such as *IL-6* and *COX-2* (red box)—which were significantly downregulated by OT—are included to illustrate potential regulatory mechanisms through which OT modulates microglial inflammatory responses. RNA-seq experiments were performed in biological triplicates (n = 3 per group). Statistical significance for DEGs was determined using DESeq2 with Benjamini–Hochberg adjusted *p* values. Abbreviations: BP, Biological Process; BV-2, BV-2 microglial cell line; CC, Cellular Component; *COX-2*, cyclooxygenase-2; DEGs, differentially expressed genes; GO, Gene Ontology; IL-17, interleukin-17; *IL-6*, interleukin-6; KEGG, Kyoto Encyclopedia of Genes and Genomes; LPS, lipopolysaccharide; MF, Molecular Function; OT, oxytocin.

Enrichment analyses of DEGs from oxytocin-pretreated BV-2 microglia showed a consistent pattern of transcriptional modulation across biological processes (BP), molecular functions (MF), cellular components (CC), and signaling pathways (KEGG) (Figure 3). In the GO BP category (Figure 3A), enriched terms were dominated by cytokine production, lipid biosynthetic process, cholesterol metabolic process, steroid biosynthetic process, and organic hydroxy compound metabolic process, indicating broad transcriptional changes across inflammatory and metabolic programs. In the MF category (Figure 3B), enrichment of lipid binding, cytokine (receptor) binding, and chemokine receptor binding pointed to coordinated modulation of receptor–ligand and lipid-associated functions. The CC category (Figure 3C) included membrane rafts/microdomains, cytoskeletal structures (stress fibers, actin filament bundles, actomyosin), and metabolic organelles (peroxisomes), showing that oxytocin-responsive genes localized to membrane, cytoskeletal, and metabolic compartments. KEGG analysis (Figure 3D) further revealed enrichment of inflammation-related pathways among OT-downregulated genes, including IL-17 signaling, as well as TNF and Toll-like receptor (TLR4) signaling. Together, these data define a coherent transcriptional signature whereby oxytocin pretreatment reshapes immune, metabolic, and structural gene networks in LPS-stimulated BV-2 microglia.

The enrichment of OT-modulated genes in this pathway suggests a targeted modulation of inflammation-related transcriptional programs by OT. A schematic representation of the IL-17 signaling pathway is shown in Figure 3E (https://www.kegg.jp/pathway/hsa04657 (accessed on 11 October 2025)), highlighting key downstream targets such as *IL-6* and *COX-2*, which were among the major OT-responsive genes identified in our dataset and significantly downregulated by OT pretreatment. In addition, a list of the top OT-downregulated DEGs—including nuclear receptor subfamily 4 group A member 1 (*Nr4a1*)—is provided in Supplementary Table S2, selected on the basis of their differential expression and their reported roles in inflammatory and microglial regulatory pathways.

## 4. Discussion

In an in vitro model of LPS-induced inflammatory response in BV2 microglia, OT pretreatment attenuated the inflammatory response, as demonstrated by transcriptomic and qPCR analyses. OT significantly reduced *IL-6* and *COX-2* expression. Moreover, differentially down-regulated genes were most significantly enriched in IL-17 signaling pathway annotation. These findings suggest that OT modulates microglial reactivity by constraining LPS-induced inflammatory transcriptional activity, with prominent reductions in *IL-6* and *COX-2*, thereby reducing pro-inflammatory transcriptional activity without global transcriptional shutdown.

In LPS-challenged BV-2 microglia, OT pretreatment downregulated *IL-6* and *COX-2* transcripts, whereas *TNF-α* showed only a modest, non-significant decrease. This pattern accords with prior reports in rodent microglial, including BV-2 lines and primary microglia—showing that exogenous OT or oxytocin-receptor agonism attenuated LPS-evoked *IL-6* and *COX-2* induction [32,33,34]. *IL-6* increases with microglial activation and contributes to neurotoxicity when sustained, and *COX-2*–dependent eicosanoid signaling sustains inflammatory circuits that compromise synaptic and neuronal integrity [45,46]. In the early phase of perinatal inflammation, *IL-6* rises rapidly across the placenta–fetal compartments tending to be higher in preterm labor. Amniotic and cervicovaginal *IL-6* levels have been linked with preterm birth and are elevated in cases of preterm labor associated with histologic chorioamnionitis. In addition, time-course studies indicate that *IL-6* peaks across maternal, placental, and fetal tissues within the first few hours after an inflammatory trigger [47,48,49,50,51]. Elevated *IL-6* is closely associated with preterm birth and early-life neuroinflammatory risk [48,52], with preterm infants showing higher cerebrospinal fluid *IL-6* levels when MRI-defined white-matter injury is present at term-equivalent age [53]. Clinical studies such as the ELGAN cohort further demonstrate that sustained postnatal inflammation, including elevated *IL-6*, correlates with long-term cognitive and motor impairments [54]. Similarly, *COX-2* is upregulated in placental and fetal tissues during chorioamnionitis and is inducible in the preterm brain following intra-amniotic LPS exposure [55,56]. Moreover, *COX-2*/Prostaglandin E2 (PGE_2_) signaling within microglia and endothelial cells disrupts oligodendrocyte maturation and contributes to white-matter injury in developing human white matter [57]. Collectively, these perinatal data together with our transcriptomic findings support an OT-associated *IL-6/COX-2* inflammatory framework that tempers microglial inflammatory tone without global transcriptional suppression [32,33,34]. This perinatal *IL-6* and *COX-2* framework should also be considered in light of the strong developmental and pathological heterogeneity of microglia. Single-cell studies show that embryonic and early postnatal microglia follow distinct maturation programs and occupy transient developmental states that differ from adult homeostatic microglia, whose identity is maintained by niche signals including transforming growth factor beta (TGF-β) and is coupled to core homeostatic functions such as tissue surveillance and support of local circuit remodeling [58,59,60]. In pathological contexts, microglia can transition into disease-associated states that diverge from homeostatic signatures, with functional shifts toward programs related to inflammatory signaling and phagocytic or lipid handling responses, and single-cell transcriptomic studies further support heterogeneous disease-associated microglial subsets across disorders [61,62]. Consistent with these state differences, recent in vivo imaging study report age-dependent differences in microglial baseline morphology and injury-response dynamics across life stages [63].

In this context, the interpretation of downstream inflammatory mediators should account for the possibility that outputs such as *IL-6* and *COX-2* are shaped by baseline microglial state and stimulus history. In line with this, primary neonatal microglia can mount robust LPS-responsive induction of *COX-2*–linked inflammatory programs in vitro [64], and primary microglia studies further support *COX-2* responsiveness to inflammatory stimulation under culture conditions [65]. In our study, OT pretreatment in a BV-2 LPS paradigm was associated with a more constrained inflammatory transcriptional response that included reduced *IL-6* and *COX-2*, providing a focused in vitro context to examine OT-sensitive inflammatory outputs. These observations support a cautious translational framing and point to primary neonatal microglia and developmental in vivo models as logical next settings to assess how OT-responsive *IL-6* and *COX-2* signals behave under age-appropriate microglial states and tissue environments.

KEGG pathway enrichment analysis identified multiple components annotated to the IL-17 signaling pathway, including *IL-6* and *COX-2*, among OT-responsive genes. The canonical IL-17 receptor A/C–ACT1 (Act1 adaptor protein)–TRAF6 (TNF receptor-associated factor 6) axis activates NF-κB (Nuclear factor kappa B), AP-1 (Activator protein 1), and MAPK signaling [66,67,68,69,70]. In the present dataset, enriched GO terms such as cytokine production, receptor binding, chemokine activity, and lipid biosynthesis indicate coordinated changes across inflammatory and metabolic programs. This GO profile suggests a cytokine-focused regulatory effect on inflammatory priming, rather than a global transcriptional suppression, with prominent changes in *IL-6/COX-2*. Our RT-qPCR data were consistent with this pattern, showing suppression of *IL-6* and *COX-2*, whereas *TNF-α* showed only a modest, non-significant decrease, rather than a generalized inhibition of TLR4-proximal responses.

Intrauterine infection/inflammation is a major driver of preterm labor, with *IL-6* serving as a key mediator at the maternal–fetal interface [71]. Increasing evidence implicates the IL-17/T helper 17 cells (Th17) axis in these processes: IL-17 concentrations are elevated in amniotic fluid, IL-17–producing cluster of differentiation 3 and 4 (CD3^+^CD4^+^) T cells accumulate in chorioamniotic membranes during preterm labor, and cord-blood Th17 signatures are enhanced in preterm neonates exposed to histologic chorioamnionitis [72,73,74]. Mechanistically, in experimental systems, IL-17A has been reported to enhance *COX-2* expression and PGE_2_ production via MAPK and AP-1 signaling, and PGE_2_ can in turn amplify IL-17 production by IL-17-producing cells, forming a feed-forward loop that may reinforce inflammatory reactivity [75,76]. Thus, our findings support a model in which OT attenuates downstream *IL-6* and *COX-2* inflammatory outputs within an IL-17/Th17-associated feed-forward inflammatory circuit, thereby limiting microglial inflammatory reactivity.

Human neonatal studies suggest that, in the absence of intrauterine inflammation, preterm infants typically exhibit lower IL-17A levels and reduced Th17 activity compared with term infants [77,78,79,80]. However, when intrauterine inflammation such as histologic chorioamnionitis is present, amplified Th17-type responses are observed. This demonstrates that the IL-17/Th17 axis is augmented in response to inflammation [3,73]. Dysregulated IL-17 signaling has been linked to multiple neonatal morbidities—including sepsis, bronchopulmonary dysplasia, patent ductus arteriosus, and necrotizing enterocolitis—highlighting its developmental relevance [13]. Moreover, IL-17A has direct neurodevelopmental effects: maternal IL-17A exposure in mice disrupts cortical morphogenesis and produces aberrant neurobehavioral phenotypes, highlighting that this pathway can strongly influence the brain during sensitive developmental windows [12,81,82]. In parallel, emerging evidence indicates that IL-17 signaling drives microglial state transitions and directly activates cortical microglia in the embryonic brain, influencing developmental circuit formation [36]. Reviews further note that microglia express IL-17RA and can proliferate, migrate, and activate in the setting of sustained IL-17A [83]. Together, these findings underscore a mechanistic framework in which OT-sensitive *IL-6/COX-2* outputs may be relevant to inflammatory contexts in which IL-17 and Th17 programs are engaged, and motivate future studies that directly quantify IL-17 ligands and receptor components in age-appropriate models.

Transcriptome profiling further revealed that OT pretreatment reduced the overall transcriptional response and markedly reduced the immediate-early transcription factor *Nr4a1*, a key regulator positioned at the AP-1–NF-κB interface [84,85] and functionally intersects with inflammatory modules that include IL-17 pathway annotations [44,68,70]. In our data, OT lowered *Nr4a1* in parallel with *IL-6/COX-2*, suggesting that OT rebalances an *Nr4a1*-centered early-response network to modulate IL-17-linked outputs without broadly suppressing the LPS program [32,34,70]. In vivo studies likewise indicate that OT selectively attenuates specific inflammatory subsets, reduces microglial activation, and preserves synaptic integrity [33,86] implicating *Nr4a1* as a key mechanistic node linking OT signaling to the IL-17-associated *IL-6/COX-2* pathway [21,87].

Some limitations should be noted. This study utilized an immortalized BV-2 cell line, which may not fully capture primary microglial phenotypes. Mechanistic specificity was inferred rather than directly demonstrated, as receptor-level engagement and targeted perturbation of the IL-17 pathway were not performed, and IL-17 ligand or IL-17 receptor components were not directly quantified at the mRNA or protein level. Furthermore, analyses focused on transcript-level changes; future work should include protein validation and functional assays of microglial behavior, including cytokine secretion at the protein level and functional readouts such as phagocytosis and migration. In addition, given that oxytocin can exert context-dependent immunomodulatory effects across tissues, including reports of NF-κB-linked inflammatory activation in human gestational tissues [88], potential undesirable pro-inflammatory effects under alternative doses, timing, routes of administration, or developmental immune states cannot be excluded. Dose–response relationships and post-treatment effects were not evaluated, and in vivo pharmacokinetics and developmental timing should be explored in follow-up studies.

## 5. Conclusions

In BV-2 microglia, OT pretreatment attenuated LPS-induced inflammatory activation, characterized by decreased *IL-6* and *COX-2* expression and suppression of IL-17-associated cytokine and chemokine modules identified through KEGG and GO enrichment analyses. These findings suggest that OT pretreatment modulates the IL-17*–IL-6/COX-2* axis rather than exerting broad transcriptional shutdown. However, because the present work is based on an in vitro BV-2 system, these findings should be interpreted as preliminary mechanistic evidence, and the functional and translational relevance of OT-mediated immunomodulation during perinatal neurodevelopment requires further validation in more complex biological models.

## Data Availability

The data presented in this study are openly available in BioProject: PRJNA1354095. [https://www.ncbi.nlm.nih.gov/sra/PRJNA1354095] (accessed on 11 October 2025).

## Supplementary Materials

The following supporting information can be downloaded at: https://www.mdpi.com/article/10.3390/life16010105/s1, Figure S1: Optimization of LPS concentration for *IBA1*; Figure S2: Validation of oxytocin effects on LPS-induced inflammatory responses in BV-2 cells; Figure S3: Quality assessment of RNA libraries; Table S1: List of primers used for quantitative real-time PCR (qPCR); Table S2: Top differentially expressed genes identified by RNA-seq.

## Abbreviations

The following abbreviations are used in this manuscript:

OT	Oxytocin
OXTR	Oxytocin receptor
IL-6	Interleukin-6
IL-17	Interleukin-17
IL-17A	Interleukin-17A
IL-17RA	Interleukin-17 receptor A
IL-17RC	Interleukin-17 receptor C
COX-2	Cyclooxygenase-2
PGE_2_	Prostaglandin E2
TNF-α	Tumor necrosis factor alpha
MAPK	Mitogen-activated protein kinase
ERK1/2	Extracellular signal-regulated kinase 1 and 2
p38 MAPK	p38 mitogen-activated protein kinase
NF-κB	Nuclear factor kappa B
AP-1	Activator protein 1
ACT1	Act1 adaptor protein
TRAF6	TNF receptor-associated factor 6
Th17	T helper 17 cells
CD3	Cluster of differentiation 3
CD4	Cluster of differentiation 4
TGF-β	Transforming growth factor beta
TLR4	Toll-like receptor 4
Nr4a1	Nuclear receptor subfamily 4 group A member 1
BV-2	BV-2 murine microglial cell line
LPS	Lipopolysaccharide
NC	Negative control
DPBS	Dulbecco’s phosphate-buffered saline
IBA1	Ionized calcium-binding adapter molecule 1
DAPI	4′,6-diamidino-2-phenylindole
IgG	Immunoglobulin G
NGS	Normal goat serum
DMEM	Dulbecco’s Modified Eagle Medium
FBS	Fetal bovine serum
bp	Base pairs
RNA	Ribonucleic acid
mRNA	Messenger ribonucleic acid
cDNA	Complementary DNA
RT-qPCR	Reverse transcription quantitative polymerase chain reaction
qPCR	Quantitative polymerase chain reaction
Ct	Cycle threshold
RNA-seq	RNA sequencing
DEGs	Differentially expressed genes
HISAT2	Hierarchical Indexing for Spliced Alignment of Transcripts 2
HTSeq-count	HTSeq read counting tool (htseq-count)
DESeq2	DESeq2 (R package for differential expression)
Ensembl	Ensembl genome database
GRCm38	Genome Reference Consortium Mouse Build 38
KEGG	Kyoto Encyclopedia of Genes and Genomes
GO	Gene Ontology
BP	Biological Process
MF	Molecular Function
CC	Cellular Component
SEM	Standard error of the mean
MA plot	M (log fold-change) vs. A (mean expression) plot
ELGAN	Extremely Low Gestational Age Newborn (study/cohort)
