# Supplementary material for: Oxytocin Modulates Microglial IL-17-Linked Inflammatory Pathways Through the IL-6/COX-2
Source: Life (Basel). 2026 Jan 12;16(1):105. doi: 10.3390/life16010105 (PMC12843464; doi:10.3390/life16010105)
Supplement: Supplementary file 1 [file life-16-00105-s001.zip › life-4035580-supplementary.pdf]

Supplementary Materials

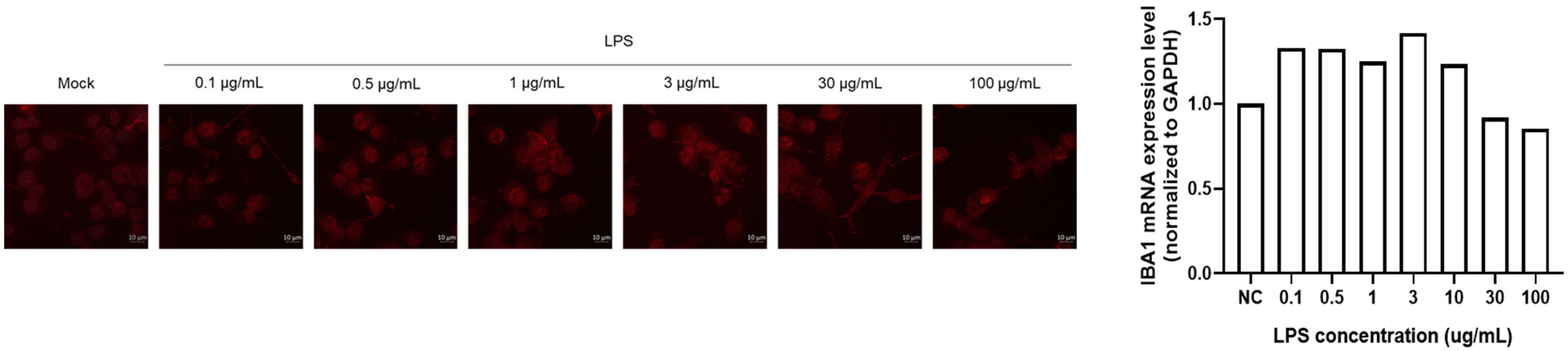

Figure S1. Optimization of LPS concentration for IBA1. Determination of LPS dose in BV-2 cells. IBA1 expression was assessed by IF and RT-qPCR. Significant induction was observed at 0.5 µg/mL LPS, which was used in subsequent experiments. Scale bar, 10 µm.

Table S1. List of primers used for quantitative real-time PCR (qPCR).

Gene	Primer	Sequence (5′-3′)
mouse GAPDH	Forward	CAT CAC TGC CAC CCA GAA GAC TG
	Reverse	ATG CCA GTG AGC TTC CCG TTC AG
mouse IBA1	Forward	TCT GCC GTC CAA ACT TGA AGC C
	Reverse	CTC TTC AGC TCT AGG TGG GTC T
mouse IL-6	Forward	TAC CAC TTC ACA AGT CGG AGG C
	Reverse	CTG CAA GTG CAT CAT CGT TGT TC
mouse TNF-a	Forward	GGT GCC TAT GTC TCA GCC TCT T
	Reverse	GCC ATA GAA CTG ATG AGA GGG AG
mouse COX-2	Forward	GCG ACA TAC TCA AGC AGG AGC A
	Reverse	AGT GGT AAC CGC TCA GGT GTT G

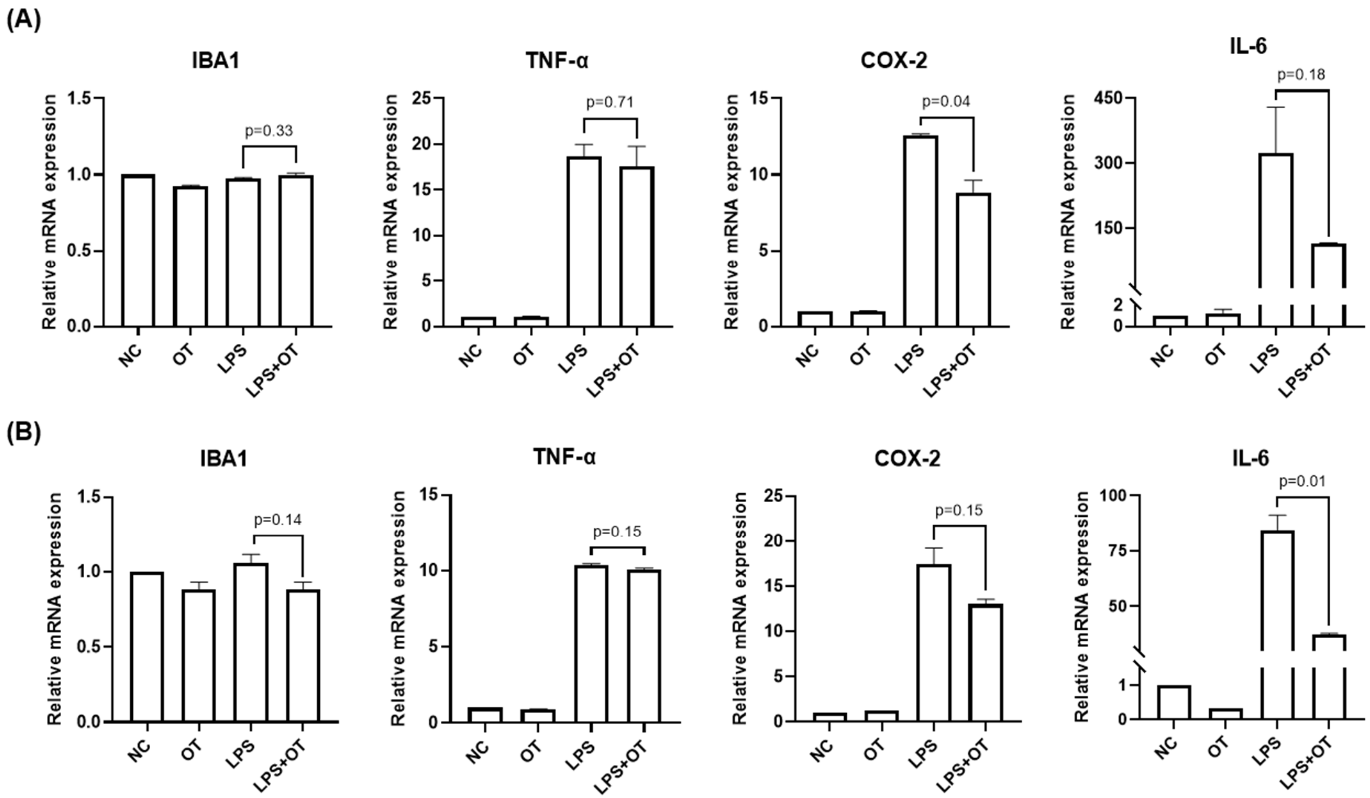

Figure S2. Validation of oxytocin effects on LPS-induced inflammatory responses in BV-2 cells.

(A) RT-qPCR analysis of IBA1, TNF-α, COX-2 and IL-6 expression under negative control (NC), oxytocin (OT), LPS, and LPS+OT conditions at 2 hours after LPS treatment. (B) RT-qPCR analysis of IBA1, TNF-α, COX-2 and IL-6 expression under the same conditions at 6 hours after LPS treatment. OT denotes oxytocin (33ng/mL). Data are mean ± SEM (n = 2), normalized to GAPDH and expressed relative to negative control. Statistical significance was determined by unpaired t-test.

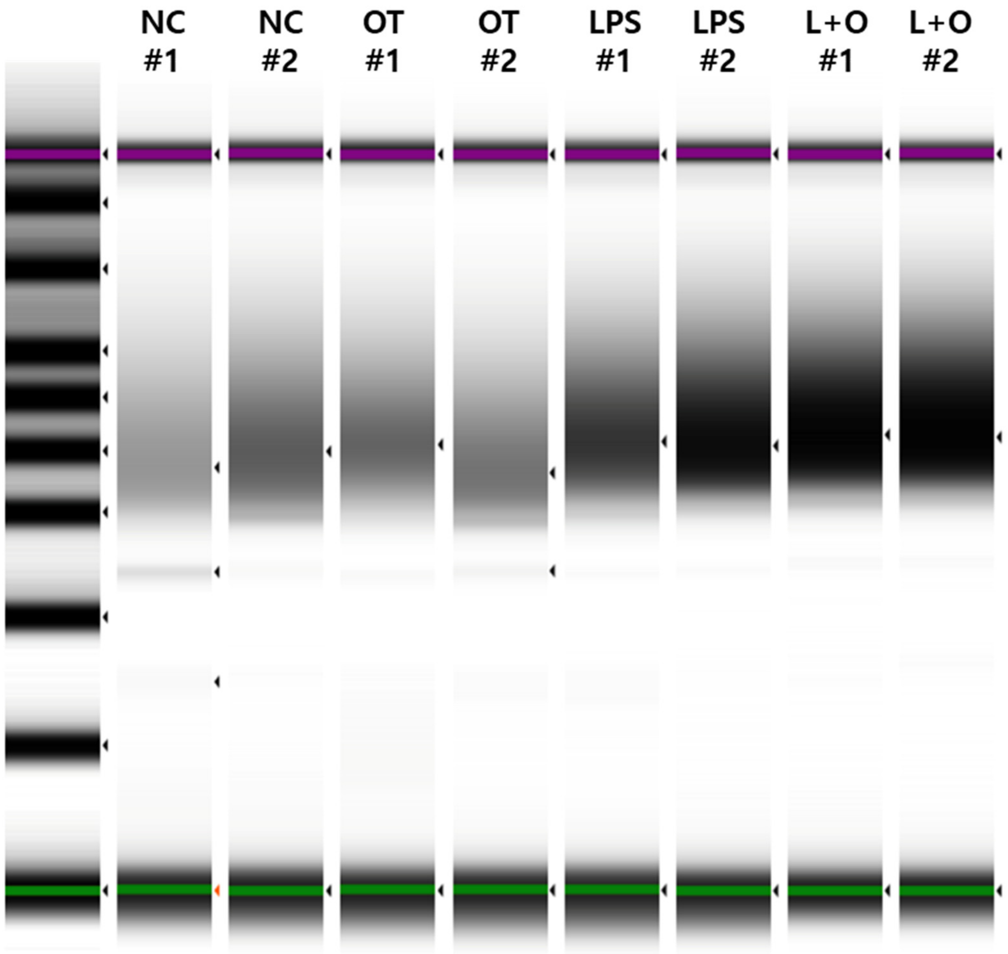

Figure S3. Quality assessment of RNA libraries. The Agilent TapeStation D1000 system electorpherogram profiles demonstrated that the libraries of all RNA samples had peaks at 200–400 bp, indicating appropriate fragment size and consistent library preparation quality for sequencing.

Table S2. Top differentially expressed genes identified by RNA-seq.

Gene	baseMean	log2FoldChange	lfcSE	stat	p-value	adj. p-value
Nr4a1	3239.677674	-1.652632869	0.077770266	-21.25018934	3.28E-100	3.47E-96
Egr3	402.9557896	-1.135199359	0.162226817	-6.997606046	2.60E-12	2.12E-09
Lbh	243.9600637	-1.030885528	0.193468842	-5.328431775	9.91E-08	2.68E-05
Klf2	387.8702587	-0.936104235	0.159121567	-5.829500086	4.03E-09	1.70E-06
Thbd	601.1613206	-0.864598897	0.159994803	-5.40391864	6.52E-08	1.97E-05
Serpine1	1738.826011	-0.748056365	0.085153989	-8.784748323	1.57E-18	2.76E-15
Hdac9	476.0156685	-0.737967542	0.148132702	-4.982103105	6.29E-07	0.000135601
Il1b	3850.985451	-1.224778119	0.079918812	-15.32523793	5.18E-53	2.74E-49
Trem1	315.3010924	-0.691031083	0.171456001	-4.003069777	5.57E-05	0.004982146
Ccrl2	424.3461072	-0.796221379	0.145475979	-5.473215457	4.42E-08	1.42E-05
